# Comparison of Subjective Health Complaints between Chinese and German University Students: A Cross-Sectional Study

**DOI:** 10.3390/ijerph121215019

**Published:** 2015-12-10

**Authors:** Janet Junqing Chu, Mobarak Hossain Khan, Heiko J. Jahn, Alexander Kraemer

**Affiliations:** Department of Public Health Medicine, School of Public Health, Bielefeld University, Universitaetsstr. 25, Bielefeld 33615, Germany; mobarak.khan@uni-bielefeld.de (M.H.K.); heiko.jahn@uni-bielefeld.de (H.J.J.); alexander.kraemer@uni-bielefeld.de (A.K.)

**Keywords:** health complaint, gastrointestinal complaints, pain/aches, perceived stress, psychological symptoms, university students

## Abstract

High rates of health complaints (HCs) with substantial variation are reported in different university populations, which can be linked to socio-demographic, lifestyle-related factors, and cultural differences. HCs can be categorized into distinct components. This study aimed to identify and compare underlying dimensions of HCs (HC components); to access and compare HC prevalence, and the associations between HC components, socio-demographic, lifestyle-related factors, and perceived stress in German and Chinese university students. Two health surveys were conducted among 5159 university students (1853 Chinese, 3306 German). Factor analysis and logistic regression were applied. The prevalence of HC ranged from 4.6% to 40.2% over the two countries. Germans reported at least three HCs more often (47.2% *vs.* 35.8%). Chinese students more often reported gastrointestinal complaints. Perceived stress was positively associated with all three HC components in both countries (OR = 1.03–1.50) with stronger associations among Germans. Women more often reported HCs (OR = 1.32–2.43) with stronger associations among the Germans. Having a father with a low educational level was associated with high psychological symptoms among the Chinese (OR = 1.51), but with low gastrointestinal complaints among the Germans (OR = 0.79). The high prevalence of HCs in students requires country-specific interventions.

## 1. Introduction

Despite the relatively young age and high educational level of university students, a high rate of health complaints (HCs) has been documented in this population [1,2]. Published studies across the globe have consistently highlighted a wide range of physical and psychological symptoms pertaining to college students. In Europe, two multi-university surveys conducted amongst university students from eight countries reported a prevalence of back pain between 35% and 43%, and nervousness between 43% and 46% [3,4]. A study conducted among 54,111 university students in the USA found a 16% depression/anxiety rate in that sample [5]. Several stressful factors could be associated with these HCs [3]. For instance, after entering university, students face new surroundings and new responsibilities. They are also responsible for handling new freedoms and opportunities. Students need to fulfill academic requirements and build up new social networks since they are away from family and friends. Some students have to cope with financial difficulties [6]. All these factors may adversely affect both physical and psychological health. The deterioration of students’ health may also affect learning ability, academic performance, goal achievement, and personal development [7]. 

Cross-national studies have demonstrated that the level of self-reported HCs varies between countries, but the patterns, or HC array, were consistent [3]. For university students, major health symptoms and complaints can be broadly categorized into relatively distinct symptom-groups, where each group comprises several complaints. Research from several countries demonstrates the coexistence of HCs, *i.e.*, reporting of one complaint significantly increases the probability of reporting one or more others. This clustering of health complaints emphasizes the need to study underlying groups, rather than a specific symptom or illness. At the same time, in order to avoid redundancy in inferential analysis (e.g., regression analysis), and to facilitate interpretations, it is useful to aggregate some of highly correlated variables to one, which represents them when possible [8]. Factor analysis can be used to reduce a large number of related variables to a smaller number of coherent factors. Previous studies in university students generally suggest two to four underlying groups of subjective HCs, such as physical conditions (e.g., pain), gastrointestinal complaints (e.g., stomach trouble), and psychological symptoms (e.g., depressive mood) [2,3]. The inequalities of HCs across different countries and populations can be linked to socioeconomic and cultural differences [2]. According to studies conducted in Europe, the associated factors to HCs and HC components in university students include demographic attributes, such as gender, age; family socio-economic status, such as income; lifestyle-related factors, such as health awareness; physical activity; nutrition awareness and perceived quality of life; and study-related stress [1,2,3,4,6]. However, there have been very few similar studies conducted in China, therefore there is a scarcity of information about China. The academic exchange between China and Germany has increased considerably in the last decade. Students of Chinese origin have become the biggest foreign student group at German universities since 2010 [9]. Under the prevailing conditions, a cross-national comparison of HCs between Germany and China is of great importance. To our knowledge, there has been no study published yet on HC prevalence comparison between Chinese and German university students. Based on the gaps mentioned above, we conducted two surveys among university students in China and Germany to achieve the following study objectives and to answer the accompanied research questions: (1) to perform factor analysis to reduce 10 self-reported HCs to smaller number HC components (accompanied research questions: Are there some underlying constructs of the HC scale? Is the factor structure between Chinese and German groups similar?); (2) to assess HC prevalence of two groups (research question: What are the prevalence rates of the HCs in the two student groups?); and (3) to compare HC prevalence, and associations between HC components and their associated factors between Chinese and German university students (research questions: Is there any significant difference in HC prevalence between two groups? Do the associations between HC components, socio-demographic, lifestyle-related factors and perceived stress differ between Chinese and German students?). This study is worthwhile. Firstly, its findings could increase the level of understanding of the extent of HCs and their associations with students’ socio-demographic and lifestyle-related characteristics. Secondly, it could be useful to tailor effective health-promoting interventions within the university settings in both countries.

## 2. Experimental Section

### 2.1. Materials and Methods

#### 2.1.1. Survey and Questionnaire 

The data used are from two studies conducted in two countries. First, a multicenter student health survey among (N = 3306) students at 16 German universities was conducted in 2006–2007 (response rate of 88%). This was followed by a Chinese student health survey at two universities in 2010–2011 (N = 1853, response rate 91%). Altogether, 5159 students from the social and natural sciences in different academic years were recruited from 18 universities in Germany and China. All universities gave permission to conduct the survey. Ethical approval for the study was obtained from the Institutional Review Board of Peking University. The students were asked to complete the survey questionnaires at the end of lectures in the lecture rooms. They were informed in writing that participation was voluntary and anonymous; they agreed to participate by completing and returning the questionnaire. No incentives were provided for participation in the survey. In both surveys, a self-administered, pre-tested questionnaire that contained questions concerning socio-demographic information, lifestyle-related attributes, a perceived stress scale, and a health complaint list including 10 items was used.

#### 2.1.2. Variables

The socio-demographic information (five variables) included gender, age, level of father’s education (“at least college level” (“high”)/“lower than college level” (“low”)), income sufficiency (“sufficient”/“insufficient”), having a partner (“yes”/“no”). 

The lifestyle-related characteristics (eight variables) included subjective general health (“good”/“poor”), health awareness (“high”/“low”), quality of life (“good”/“poor”), importance of nutrition (“important”/“unimportant”), importance of good grades at university (“important”/“unimportant”), alcohol consumption (“never”/“less than once a week”/“at least once a week”), physical activity (“less than once a week”/“once to twice a week”/“at least three times a week”) and BMI (calculated from self-reported weight and height using Metric BMI Formula: BMI = weight in kilograms divided by the square of height in meters). 

Perceived stress was assessed, using Cohen’s Perceived Stress Scale (PSS) [10], to ascertain the extent to which participants appraised life situations over the previous month as stressful. The scale yielded a single score, with higher scores indicating higher levels of stress.

Additionally, 10 different physical and psychological HCs adopted from previous studies [2,4] were investigated (neck/shoulder pain, back/low back pain, headaches, stomach trouble, diarrhea, constipation, concentration difficulties, nervousness/anxiety, mood swings, and depressive mood). Respondents were asked to report how often they had experienced each specific HC during the previous year on a 4-point Likert-type scale (“never”, “rarely”, “quite often”, “very often”). We recoded the responses into a dichotomous variable (“never”/“rarely” = “seldom” *vs.* “quite often”/“very often” = “often”) in order to present and compare HC prevalence between countries. Cronbach’s alpha of the HC scale was 0.79 for the Chinese subsample and 0.74 for the German subsample.

#### 2.1.3. Statistical Analysis 

Frequency and cross tabulation analyses were conducted to describe the characteristics of the sample, and to compare the prevalence rates of HCs *often* experienced between Chinese and German students. In order to combine the 10 HC items into appropriate components, a factor analysis (using principle component analysis, PCA) was applied. Three criteria suggested that the data were suitable for PCA: (1) the sample size of 5159 respondents was larger than the minimum 300 recommended for PCA, (2) the ratio of respondents to items of 469 to 1 was higher than the suggested 10 to 1 ratio [8], and (3) the correlation matrices produced for the 10 HC items showed many statistically significant coefficients above 0.3–0.5 (data not presented). The component correlations were above 0.3 for both groups, therefore, Oblimin rotation with Kaiser Normalization was performed to reduce 10 HC items into three HC components [8]. For the subsequent analysis, we calculated a sum score for each of the three HC components, which were then divided by the number of items in each component. For multivariable binary logistic regression, first these scores were dichotomized by median split (high prevalence *vs.* low) to consider as dependent variables. Then, we assessed the associations of each dichotomized HC component with socio-demographic and lifestyle-related factors as well as with perceived stress for each country separately. Since the PCA showed the eigenvalue for the third HC component (pain/aches) for the Chinese group was slightly smaller than 1 (0.94), the amount of information captured by this HC component from the Chinese subsample may be less than the information contained in the original set of variables [8]. Therefore, supplementary multivariable logistic regression analyses were conducted to assess the factors associated with the HCs in the pain/ache group *i.e.*, neck/shoulder pain (often *vs.* seldom), back/low back pain (often *vs.* seldom) and headaches (often *vs.* seldom) for the Chinese group additionally. The analysis was performed using IBM SPSS statistics 21. For all tests, the significance level was set at *p* < 0.05.

## 3. Results

### 3.1. General Characteristics of the Sample

The sample description is presented in Table 1. In general the German sample was older, with more females and higher BMI. In both countries, the majority of students rated their general health as good. Chinese students reported a higher rate of sufficient income, a lower rate of good quality of life, and consumed alcohol less frequently than their German counterparts. 

**Table 1:** Sample description of the university students in China and Germany.

Variables		%		^†^ *p*-Value
		Chinese (N = 1853)	German (N = 3306)	
Gender	Male	52.1	47.3	0.001
	Female	47.9	52.7	
Father’s education	High	45.8	52.8	<0.001
	Low	54.2	47.2	
Having a partner	Yes	31.4	56.0	<0.001
	No	68.6	44.0	
Income sufficiency	Sufficient	81.2	58.5	<0.001
	Insufficient	18.8	41.5	
Subjective general health	Good	89.2	87.0	0.025
	Poor	10.8	13.0	
Health awareness	High	67.2	45.5	<0.001
	Low	32.8	54.5	
Nutrition importance	Important	89.3	91.8	0.003
	Unimportant	10.7	8.2	
Grade importance	Important	92.8	96.2	<0.001
	Unimportant	7.2	3.8	
Physical activity	≥ 3 times a week	22.7	41.3	<0.001
	1–2 times a week	48.8	36.2	
	Less than once a week	28.5	22.5	
Alcohol consumption	Never	50.4	9.8	<0.001
	Less than once a week	40.6	30.4	
	At least once a week	9.0	59.8	
Life quality	Good	39.0	57.8	<0.001
	Poor	61.0	42.2	
		Median (quartile deviation)		
Age		21.0 (1.5)	23.0 (2.0)	<0.001
BMI		20.1 (1.6)	22.3 (1.9)	<0.001

Note: **^†^** Chi square test for categorical variables and Mann-Whitney-U-test for continuous variables.

### 3.2. Data Dimension Reduction

Factor analysis of the 10 HCs provided three HC components. The presence of three components explained about 57% of the total variance for both Chinese and German groups. Table 2 describes these three HC components (psychological, pain/aches, gastrointestinal) as well as the related item loadings. 

**Table 2:** Factor analysis of 10 self-reported HCs reduced to three HC components.

Health Complaint	Chinese			German		
	Components			Components		
	1	2	3	1	2	3
	Psychological (4 Items)	Gastrointestinal (3 Items)	Pain/aches (3 Items)	Psychological (4 Items)	Pain/Aches (3 Items)	Gastrointestinal (3 Items)
Component eigenvalue	3.37	1.19	0.94	2.99	1.29	1.20
Percentage of variance explained by component	36.45%	12.18%	9.36%	31.89%	13.19%	12.13%
Depressive mood	0.85			0.84		
Mood swings	0.79			0.84		
Nervousness/anxiety	0.79			0.71		
Concentration difficulties	0.66			0.56		
Back/low back pain			−0.87		0.87	
Neck/shoulder pain			−0.73		0.86	
Headaches			−0.36		0.53	
Diarrhoea		0.77				0.82
Constipation		0.51				0.71
Stomach trouble		0.72				0.59

Notes: Oblimin rotation method was used; the Kaiser-Meyer-Olkin (KMO) value for Chinese was 0.84, for German was 0.75; the Barlett’s test of sphericity value was significant (*p* < 0.001) for both Chinese and German.

### 3.3. Prevalence and Number of Health Complaints Reported in the Previous Year

The country-specific prevalence of HCs (divided into three categories) and their comparisons between the two countries are presented in Table 3. Many students in both China and Germany reported psychological symptoms and pain/aches. More German students reported multiple HCs than Chinese students during the previous year. 

**Table 3:** Comparisons of HC prevalence between Chinese and German university students.

Health Complaints (Often)		Prevalence (%)		
		Chinese	German	Chi Square Test *p*-Value
Total HCs	Seldom HCs	31.8	19.5	<0.001
	One to two HCs	32.4	33.3	
	At least three HCs	35.8	47.2	
Psychological HCs	Seldom psychological HCs	48.0	37.4	<0.001
	One to two psychological HCs	34.7	43.6	
	At least three psychological HCs	17.3	19.0	
Concentration difficulties		27.8	40.2	<0.001
Nervousness/anxiety		27.3	38.3	<0.001
Mood swings		33.1	30.1	0.028
Depressive mood		18.7	16.3	0.025
Pain/aches	Seldom pain/aches	58.0	40.9	<0.001
	One to two pain/aches	36.8	46.5	
	Three pain/aches	5.2	12.6	
Neck/shoulder pain		26.1	37.0	<0.001
Back/low back pain		20.8	36.9	<0.001
Headaches		19.0	31.9	<0.001
Gastrointestinal HCs	Seldom gastrointestinal HCs	69.0	74.2	<0.001
	One to two gastrointestinal HCs	29.4	25.1	
	Three gastrointestinal HCs	1.6	0.7	
Stomach trouble		12.7	17.3	<0.001
Diarrhoea		12.7	11.6	0.250
Constipation		15.5	4.6	<0.001

**Table 4:** Odds ratio (OR) and 95% confidence interval (95% CI) for three health complaint component groups (high prevalence *vs.* low) by perceived stress, socio-demographic and lifestyle-related characteristics.

Variables		Psychological OR (95% CI)		Pain/aches OR (95% CI)		Gastrointestinal OR (95% CI)	
		Chinese ^†^ R^2^ = 0.33 (N = 1476)	German R^2^ = 0.33 (N = 2838)	Chinese R^2^ = 0.15 (N = 1467)	German R^2^ = 0.18 (N = 2880)	Chinese R^2^ = 0.09 (N =1474)	German R^2^ = 0.09 (N = 2881)
Gender	Male (^‡^ Ref.)	1.18 (0.93–1.50)	1.68 *** (1.38–2.04)	1.32 * (1.05–1.67)	2.43 *** (2.04–2.89)	0.97 (0.78–1.20)	1.65 *** (1.36–1.99)
	Female						
Father’s education	High (Ref.)	1.51 ** (1.17–1.93)	0.91 (0.77–1.09)	0.88 (0.69–1.12)	1.00 (0.85–1.17)	1.08 (0.86–1.34)	0.79 ** (0.67–0.94)
	Low						
Having a partner	Yes (Ref.)	0.94 (0.72–1.22)	1.12 (0.94–1.34)	0.92 (0.71–1.19)	0.75 *** (0.64–0.88)	0.94 (0.74–1.19)	0.78 ** (0.65–0.92)
	No						
Income sufficiency	Sufficient (Ref.)	0.96 (0.70–1.32)	1.31 ** (1.09–1.57)	1.35 * (1.01–1.82)	1.18 * (1.01–1.40)	1.30 (0.97–1.72)	1.13 (0.95–1.35)
	Insufficient						
Subjective general health	Good (Ref.)	1.96 ** (1.30–2.96)	1.50 ** (1.14–1.98)	2.66 *** (1.84–3.85)	3.02 *** (2.29–3.99)	1.76 ** (1.22–2.53)	1.44 ** (1.13–1.84)
	Poor						
Health awareness	High (Ref.)	1.18 (0.91–1.52)	0.86 (0.72–1.04)	0.97 (0.76–1.24)	0.95 (0.80–1.12)	1.11 (0.89–1.40)	1.10 (0.91–1.32)
	Low						
Physical activity	Less than once a week	1.12 (0.79–1.60)	1.24 (0.98–1.58)	1.37 (0.97–1.92)	1.53 *** (1.23–1.91)	1.16 (0.85–1.58)	1.21 (0.96–1.53)
	Once to twice a week	1.01 (0.74–1.38)	1.12 (0.91–1.36)	1.21 (0.89–1.63)	1.13 (0.94–1.35)	1.15 (0.87–1.50)	1.21 (0.99–1.47)
	At least three times a week (Ref.)						
Alcohol consumption	Never (Ref.)						
	Less than once a week	1.14 (0.89–1.48)	1.02 (0.73–1.42)	1.27 (0.99–1.62)	0.85 (0.63–1.14)	0.89 (0.71–1.12)	0.85 (0.63–1.15)
	At least once a week	1.04 (0.65–1.68)	1.19 (0.87–1.63)	1.97 ** (1.26–3.09)	0.94 (0.70–1.24)	1.09 (0.71–1.68)	0.79 (0.59–1.05)
Nutrition importance	Important (Ref.)	1.35 (0.88–2.08)	1.11 (0.80–1.53)	1.50 * (1.01–2.22)	1.00 (0.74–1.35)	1.14 (0.77–1.67)	1.01 (0.73–1.40)
	Unimportant						
Grade importance	Important (Ref.)	0.62 (0.37–1.04)	1.12 (0.69–1.80)	0.73 (0.45–1.20)	0.61 * (0.40–0.95)	0.60 * (0.39–0.94)	0.96 (0.61–1.50)
	Unimportant						
Life quality	Good (Ref.)	1.34 * (1.03–1.75)	1.66 *** (1.37–2.00)	1.08 (0.83–1.40)	1.29 ** (1.08–1.54)	1.09 (0.86–1.39)	1.29 ** (1.07–1.56)
	Poor						
Age per year increase		0.97 (0.92–1.03)	1.03 * (1.01–1.05)	0.95 (0.90–1.01)	1.00 (0.98–1.02)	1.05 (0.99–1.10)	1.03 * (1.01–1.05)
BMI per point increase		0.96 (0.92–1.01)	0.97 * (0.94–0.99)	1.01 (0.96–1.05)	1.00 (0.98–1.03)	0.92 *** (0.88–0.96)	1.02 (1.00–1.05)
PSS score per point increase		1.15 *** (1.13–1.18)	1.50 *** (1.43–1.57)	1.07 *** (1.05–1.09)	1.11 *** (1.07–1.16)	1.04 *** (1.03–1.06)	1.11 *** (1.07–1.16)

Notes: ^†^ In all regression models Nagelkerke R^2^ was reported, df was 16; ^‡^ Reference category; Significance of Wald test: * *p* < 0.05; ** *p* < 0.01; *** *p* < 0.001.

### 3.4. Factors Associated with Health Complaints

Multivariable logistic regression models (Table 4) showed the associations between a high frequency of three HC components (dependent variables) and socio-demographic, lifestyle-related factors, as well as perceived stress by country. The summary of additional multivariable logistic regression models for the Chinese students with neck/shoulder pain (often *vs.* seldom), back/low back pain (often *vs.* seldom) and headaches (often *vs.* seldom) is presented in Table 5. Perceived stress was positively associated with all three HC components in both countries, with stronger associations in German students. Among German students having no boy/girlfriend was related to less often pain/aches and gastrointestinal complaints. Females reported more frequent HCs in all three groups among German students. Having a father with a low educational level was related to more frequent psychological HCs among the Chinese but associated with less frequent gastrointestinal HCs among the Germans. For the Chinese students, most independent variables included in the analyses showed similar association with high frequency of pain/ache component and individual HCs of the pain/ache group. Noteworthy aspects are paying less attention to nutrition revealed positive association only with often headaches, less physical activity were associated with neck/shoulder pain, and no gender difference was found in pain/ache group among the Chinese students. 

**Table 5:** Odds ratio (OR) and 95% confidence interval (95% CI) for three pain/aches (often *vs.* seldom) by perceived stress, socio-demographic and lifestyle-related characteristics in Chinese students.

Variables		Neck/Shoulder Pain	Back/Low Back Pain	Headaches
		^†^ R^2^ = 0.11 (N = 1481)	R^2^ = 0.09 (N = 1475)	R^2^ = 0.15 (N = 1479)
Gender	Male (^‡^ Ref.)	1.19 (0.93–1.52)	1.26 (0.97–1.64)	1.30 (0.98–1.75)
	Female			
Father’s education	High (Ref.)	0.96 (0.75–1.24)	0.73 * (0.56–0.97)	1.09 (0.81–1.48)
	Low			
Having a partner	Yes (Ref.)	0.92 (0.70–1.20)	0.91 (0.68–1.22)	0.64 ** (0.47–0.87)
	No			
Income sufficiency	Sufficient (Ref.)	1.37 * (1.01–1.87)	1.51 * (1.08–2.09)	1.69 ** (1.19–2.39)
	Insufficient			
Subjective general health	Good (Ref.)	1.84 ** (1.28–2.65)	2.29 *** (1.57–3.33)	2.74 *** (1.86–4.06)
	Poor			
Health awareness	High (Ref.)	0.91 (0.70–1.18)	0.99 (0.75–1.32)	1.12 (0.83–1.53)
	Low			
Physical activity	Less than once a week	1.47 * (1.02–2.12)	1.25 (0.85–1.83)	1.07 (0.71–1.61)
	Once to twice a week	1.43 * (1.03–1.98)	0.98 (0.70–1.39)	0.75 (0.52–1.10)
	At least three times a week (Ref.)			
Alcohol consumption	Never (Ref.)			
	Less than once a week	0.92 (0.71–1.19)	1.21 (0.91–1.61)	1.22 (0.89–1.68)
	At least once a week	1.51 (0.95–2.42)	2.47 *** (1.54–3.96)	3.38 *** (2.05–5.57)
Nutrition importance	Important (Ref.)	1.39 (0.92–2.08)	1.33 (0.87–2.03)	1.97 ** (1.27–3.05)
	Unimportant			
Grade importance	Important (Ref.)	0.63 (0.36–1.10)	1.05 (0.62–1.77)	0.96 (0.53–1.72)
	Unimportant			
Life quality	Good (Ref.)	1.07 (0.81–1.42)	1.16 (0.85–1.58)	0.92 (0.66–1.29)
	Poor			
Age per year increase		0.94 (0.89–1.00)	0.96 (0.90–1.03)	0.92 * (0.85–0.99)
BMI per point increase		0.99 (0.94–1.04)	0.99 (0.94–1.04)	0.99 (0.94–1.05)
PSS score per point increase		1.06 *** (1.04–1.08)	1.03 ** (1.01–1.05)	1.06 *** (1.04–1.08)

Notes: ^†^ In all regression models Nagelkerke R^2^ was reported, df was 16; ^‡^ Reference category; Significance of Wald test: * *p* < 0.05; ** *p* < 0.01; *** *p* < 0.001.

## 4. Discussion

This cross-national study assessed the underlying dimensions (*i.e.*, HC components) and prevalence of HCs, and their relation with perceived stress, socio-demographic, and lifestyle-related characteristics in Chinese and German university students. Such information is important for guiding the selection of intervention strategies at universities in the two countries. The current study had three specific objectives with five accompanied research questions.

### 4.1. Assessment of Underlying Dimensions of the 10 HCs through Factor Analysis 

The answer to our first research question regarding the underlying dimensions of the HC scale is positive. In agreement with a study conducted among university students from Germany, Spain and Lithuania (containing nine symptoms) [2], our HC scale was categorized into three components: psychological symptoms; gastrointestinal complaints; and pain/aches. Because the items associated with the three components appear similar to one another and make theoretical and logical sense, it indicates there are coherent constructs of the HC scale in our sample [8]. Regarding the second research question of the factor structure comparison between the two groups, 10 HC item responses load on the same constructs (components) across two groups. The total variance explained by the three components, and the factor loadings are similar for two groups, apart from loadings on the pain/ache component. This may be due to the low eigenvalue for the pain/ache component for the Chinese group. Low variance explained by this component in the Chinese may be related to the relatively low prevalence of pain/ache among the Chinese compared to the Germans (19.0%–26.1% *vs.* 31.3%–37.0%). Although three components explain similar amount of total variance for Chinese and German groups, the percentage of variance explained by each component is different between the two groups. These results may also imply the influence of cultural backgrounds on patterns of reporting HCs across university students [11]. Further research in this regard in Chinese as well as in students from other cultures is granted.

### 4.2. Prevalence of Health Complaints

Concerning the third research question about HC prevalence rates of our sample, we found that more than one third of the Chinese students (35.8%) and almost half of the Germans (47.2%) had experienced three or more HCs during the previous year. The findings (17.3% of Chinese and 19.0% of German students with at least three psychological symptoms and 5.2% Chinese and 12.6% of Germans with all three pain/aches) suggest that having multiple HCs is frequent among university students. Such patterns of potential “cluster symptoms” might be expected, given the tendency for specific healthy/unhealthy lifestyle factors to aggregate in clusters in university students [12]. For instance, Kindermans *et al*. found that avoidance of physical activity as a result of chronic pain can lead to subsequent emergence of more symptoms, such as depressive mood [13]. Nevertheless, compared with previous studies, the multiple HC prevalence in our sample is relatively low. For example, El Ansari *et al.* found that 53.0% and 85.7% of the British and Egyptian students reported ≥3 psychological symptoms, while 38.0% and 68.7% of them reported at least three pains/aches, respectively [3,6]. 

The array of symptoms across our sample agrees with the findings of previous studies among university students in other countries, which used a similar scale [3,4,6]. For instance, Stock *et al.* reported that nervousness (46%), headache (37%), back ache (35%), neck/shoulder pain (34%), and depressive mood (25%) were the most frequently reported complaints in a study conducted in seven European countries [3]; while El Ansari *et al.* found the symptoms most often reported among students from seven universities in the UK were headache (59.5%), concentration difficulties (54.5%), back pain (43.3%), nervousness/anxiety (43.2%), mood swings (42.1%), and neck/shoulder pain (39.4%) [4]. In our sample, the most frequently reported symptoms in the previous 12 months in both China and Germany were in the psychological group: concentration difficulties, nervousness/anxiety, mood swings; and in the pain/aches group: neck/shoulder pain, back/low back pain, and headaches. These findings strongly suggest the dominant role of psychological complaints and musculoskeletal problems among subjective health complaints in university students. The wide use of computers and other electronic equipment at universities could be a cause of such complaints [2], because experts agree that excessive keyboard use, particularly with the wrist and elbow in awkward positions, probably plays a role in the development of complaints of the arm, neck and shoulder [14,15]. 

### 4.3. Health Complaints and Associated Factors

As an answer to our fourth research question referring to HC prevalence comparison, German students reported a higher number of HCs related to psychological symptoms (except mood swings and depressive mood) and all pain/aches, but a lower number of gastrointestinal complaints (except stomach trouble) than their Chinese counterparts. Our findings of HC prevalence in German students are comparable with the results for German students in Stock *et al.*, except that the prevalence of pain/aches in the Stock *et al.* study was slightly higher [3]. However, compared with the students in Egypt, Libya and the UK, our German sample had a lower prevalence in almost every HC investigated (the exception being a similar frequency of back/low back pain as that of the UK and Libyan students) [4,6,16]. Chinese students reported a far lower prevalence of every HC than students from Libya and eight European countries apart from a comparable frequency of constipation with the students from the UK [3,4,6,16]. It is unclear why differences in health complaints among student populations exist. However such discrepancies could result from true differences in the prevalence of these problems, or from cultural differences in thresholds for reporting particular kinds of problems [1]. Differences in language and vocabulary may also influence the expression of symptoms, because some countries may have several synonyms for one or more complaints [17].

With respect to our fifth research question on HC prevalence associated factors, we found common associated factors with HC components in two groups, such as a positive association between perceived stress, poorly rated general health and all three HC components. We also found different associated factors between two groups, such as gender difference in terms of women reporting more HCs among the Germans, a connection between alcohol consumption and back/low back pain and headaches among the Chinese. At the same time, we also identified same factor with different associations in two groups, such as a higher BMI was related to less psychological symptoms among the Germans, but less gastrointestinal complaints among the Chinese; while having a father with a low educational level was related to more psychological symptoms in the Chinese students but less gastrointestinal complaints in the German students.

In line with a number of other studies, we found that a high level of perceived stress was independently associated with all three HC components with the strongest association being with psychological symptoms [2,3,17,18,19]. Our findings were also in agreement with previous findings that greater physical activity, better nutrition, stress management training, and low screen time were negatively correlated with psychological HCs such as anxiety, and depressive mood among university students [20,21]. Our finding that BMI was negatively associated with gastrointestinal complaints among Chinese students agree with findings of previous studies, conducted among students in Japan and China, that young female students had a greater desire to be thinner despite the very low overweight prevalence of this population (<3%); their restrict consumption of certain foods, more alcohol consumption, and irregular lifestyle were significantly related to health complaints [22,23,24]. These findings provide information about the connection between lifestyle-related behaviors and health complaints among university students. Steptoe and Wardle suggest that an unhealthy lifestyle associated with lack of information about health and behavior, and greater beliefs in uncontrollable influences may contribute to poor health in students. Meanwhile, change in health behavior, and the adoption of effective coping strategies involve not only the availability of the relevant knowledge to the individual but also a supportive institutional infrastructure which promotes the change [25]. A poll among students in Hong Kong found that most participants knew that eating adequate amount of fruit and sufficient sleep are essential for maintaining health, but over 60% of students failed to eat fruit on a daily basis, and only one-quarter of them got enough sleep [26]. In order to enable the students to take charge of personal health responsibilities, take part in physical activities, and to maintain good nutritional habits, universities have the responsibility to provide appropriate resources for healthy lifestyle knowledge and activities; for example, integrating programs on how to cope with stress into the curriculum and providing more fresh food on campus. 

Our finding that grade importance was associated with physical HCs in both Chinese and German students agrees with previous studies, which suggest the role of school-related stress in the development and maintenance of such health complaints [3,27,28]. At the same time, cultural difference may have influence on the prevalence of HCs in students. We found that having a father with a low educational level was associated with a higher chance of reporting psychological HCs in China, but fewer gastrointestinal HCs in Germany. This may reflect the big disparity in Chinese society between people with and without a higher education degree. It has been reported that educational attainment has a strong positive effect on self-rated health in China, while the socioeconomic disparity in health is bigger in mainland China than it is in Western countries [29,30]. In contrast to the findings of Stock *et al.*, that alcohol use and problem drinking were positively associated with the frequency of psychological HCs but negatively with pain [4], we found that alcohol consumption was positively associated with back/low back pain and headaches among Chinese students, but no connection between HCs and alcohol use was revealed among German students. A possible explanation for these findings could be that we used once a week as the measure for drinking due to the low alcohol consumption in general in our Chinese sample; this amount of alcohol might not be sensitive enough for the German students, since the majority of them (60%) reported drinking at least once a week. Consistent with Edwards *et al.*, who reported that negative social exchanges (events such as “lost temper”) were a significant predictor of physical symptoms [31], we found having a boy/girlfriend was related to more frequent physical HCs among Germans. Effective interventions for such students may need to include relationship counseling and training in communications skills. In our study, the gender difference in terms of females reporting HCs more often was revealed among Germans. Previous studies could not separate the factors for gender difference from biological factors, acquired risks, psychosocial aspects and health reporting behavior [3,4,28]. Despite the Chinese tradition of son-preference, under the One-child Policy implemented in China since 1980s, the Chinese parents pledge themselves to foster their only-child regardless of the child’s gender. The only-child generation girls have gained more chances in personal development, education, and promotion in the society. Based on Katz and Boswell, the gender gap was found reduced among only-children as compared to non-only children, *i.e.*, with only-child girls behaving more like boys [32]. According to Torsheim *et al*. the gender differences in health complaints may reflect the influence of gender role development in the society. A modern gender role development would make girls less susceptible to report health complaints, and boys more prone to report health complaints. It has been reported in countries where more gender empowerment exists; not only girls, but also boys had a lower level of health complaints [28]. In our study, the high rate (66%) of only-children among the Chinese sample may explain the results that relatively low HC prevalence and no gender difference in HCs was found in this group to a certain extent. 

In line with a number of studies conducted among university students in different countries, we found that perceived poor general health was positively associated with frequent HCs in both Chinese and Germans. Better quality of life was associated with lower frequency of all three groups of HCs in Germans [3,4,6,16]. It is possible that poorly rated general health, along with self-reported HCs are often mirrored in unfavorable ratings of one’s quality of life. Linardatou *et al.* reported that participants in stress management programs displayed significantly fewer psychological symptoms and an improved perception of life quality [33]. This may highlight the importance of providing integrated stress management programs at universities in both countries. 

Limitations of this study include the following: (1) Although a representative sample of students was sought at the universities by selecting courses that represented the different departments/faculties, due to organizational difficulties and variations in the response rate, students of health sciences were overrepresented in the final sample in both countries. Therefore, even with the big sample and good response rates, our sample remains a convenience sample, and generalizations of the findings should be made with caution. On the other hand, it was beyond the researchers’ capacity to select representative sample of university students for the two countries, especially for a huge country like China that reveals remarkable economic and developmental differences among regions. We chose two comprehensive universities (one is from the south, another from the north) that enrol students from all over the country. Nevertheless they cannot be generalised to all Chinese universities due to their great renown; (2) As data were self-reported, they may be subject to sources of error e.g., recall bias and social desirability; (3) Students were recruited during lectures; hence, those were not present in the class during data collection were not included in the survey. This may have affected the results since absence from lectures might be due to psychological or physical health problems; (4) Due to the different data collection periods in the two countries, period effect cannot be excluded; (5) Since different perceived stress scales were applied (PSS-14 in China, PSS-4 in Germany), a direct score comparison of perceived stress between the two countries was not possible; (6) As a cross-sectional survey, the findings are associations not causations; for instance, it is possible that stress is the cause of the HCs, or the HCs could cause the stress. 

Finally, it is noteworthy, that the following differences between Chinese and German educational systems may also have influence on the comparability of the two countries. In China, high-school graduates enter college at the end of the 12th grade, in Germany after the 13th year (before 2011). The enrolment rate of high-school graduates in Germany was 39.8% in 2007, in China was 23.3% in 2008 [34,35]. University admission in China depends solely on students’ grade on the College Entrance Examination, while existing disparities between regions and ethnic groups could have influence on their achievement with the test [35]. In Germany, determinants of attending university include social background of parents, students’ expectation on receiving student loan and employment probability after the study [36].

## 5. Conclusions

Despite some limitations, several important conclusions can be drawn from our research. Firstly, many students are experiencing one or more HCs and the high prevalence of HCs such as concentration difficulties, nervousness and pain indicate a need for preventive action at universities. Secondly, the relationship between perceived stress, lifestyle-related characteristics and HCs provides valuable information for tailoring intervention strategies at Chinese and German universities. For instance, providing relationship counseling for the German students, and helping to shape a realistic body image and reducing alcohol consumption in the Chinese may be targeted intervention measures. Thirdly, the results provide specific information for educators in other countries to aid in understanding the HCs of students from China and Germany, especially when considering the fact that Chinese students have become the biggest foreign student group in the English-speaking world [37]. 
